# Impact of Patellar Tendinopathy on Isokinetic Knee Strength and Jumps in Professional Basketball Players

**DOI:** 10.3390/s21134259

**Published:** 2021-06-22

**Authors:** Marc Dauty, Pierre Menu, Olivier Mesland, Bastien Louguet, Alban Fouasson-Chailloux

**Affiliations:** 1 CHU Nantes, Physical Medicine and Rehabilitation Center, University Hospital of Nantes, Hôpital St Jacques, 85 Rue Saint Jacques, 44093 Nantes, France; marc.dauty@chu-nantes.fr (M.D.); pierre.menu@chu-nantes.fr (P.M.); 2 CHU Nantes, Service de Médecine du Sport, University Hospital of Nantes, Hôpital St Jacques, 85 Rue Saint Jacques, 44093 Nantes, France; olivier.mesland@chu-nantes.fr (O.M.); bastien.louguet@chu-nantes.fr (B.L.); 3 INSERM UMR U1229 RMeS, Regenerative Medicine and Skeleton, Nantes University, 44000 Nantes, France; 4 IRMS, Institut Régional de Médecine du Sport, Hôpital St Jacques, 85 Rue Saint Jacques, 44093 Nantes, France

**Keywords:** knee, tendon, muscle, sport, isokinetic, performance

## Abstract

Patellar tendinopathy is characterized by tendon pain which may reduce the level of performance. This study’s main aim was to compare isokinetic knee strength and jump performances at the start of the sport season between players with patellar tendinopathy and those without. Secondary aims were to assess the relationship between knee strength and jump function. Sixty-two professional basketball players were enrolled (mean age: 25.0 ± 4.0). All players performed knee isokinetic measurements, single leg countermovement jumps, and one leg hop tests. Correlations between knee strength and jump performances were examined. Twenty-four players declared a patellar tendinopathy and were compared to the 38 players without tendinopathy. The isokinetic quadriceps strength was lower in cases of patellar tendinopathy, and a camel’s back curve was observed in 58% of the cases of patellar tendinopathy. However, jump performances were preserved. No link was found between quadriceps and hamstring limb symmetry indexes at 60 and 180°/s with jumps. This preseason screening enabled us to identify the absence of consequences of patellar tendinopathy in professional basketball players. Jump performances were not altered, possibly due to compensatory strategies.

## 1. Introduction

Patellar tendinopathy (PT) is characterized by serious tendon pain, which may reduce level of competitive performance; it also induces loss of playing time and may cause premature career endings [1,2]. PT prevalence and incidence in basketball players are estimated at 32% and 22%, respectively [3,4]. Depending on the severity of symptoms, 30% of the athletes affected by severe PT do not return to sport, with 50% of them still having knee pain 15 years after the diagnosis [5]. In other cases, return-to-sport is possible after a time loss of 3 to 14 days [4].

Sports with repetitive jumps represent a particular cause of PT [6,7,8,9]. However, different other risk factors have been described, such as high bodyweight, high BMI or high leg-length, a low foot arch, low quadriceps and hamstring flexibility and low relative quadriceps strength [10]. More recently, only high bodyweight and high countermovement jump were considered risk factors, but vertical jumps were not [11]. Actually, the concept of risk factors established from cross-sectional or case–control studies is confusing. Indeed, according to Van Mechelen’s model of injury prevention, only prospective studies can be predictive [12]. Thus, all the previously cited parameters cannot be confirmed as risk factors [2,13,14]. A decrease in knee strength or vertical jump performances in basketball players should more accurately be considered a consequence of PT or of another knee joint injury than a risk factor [15,16]. The identification of PT in professional basketball players is often difficult because players are used to playing with tendon pain without complaining, for fear of not advancing in competition [17,18]. As such, it is an important challenge to identify the risk of lower limb injury, such as anterior cruciate ligament tear [19], thigh muscular injuries [20] and PT. As PT could be responsible for limitations in sport performances, isokinetic testing and jump evaluations could be relevant in its identification [15], especially because preventive programs already exist to limit PT consequences [21].

Based on this observation, the main objective of this study was to compare isokinetic knee strength and jump performances at the start of the sport season between players with PT and those without to confirm the interest of these evaluations. Secondary aims were to assess the relationship between knee strength and jump function, and to identify risk factors of patellar tendinopathy.

## 2. Methods

### 2.1. Participants and Recruitment

All basketball players of three professional teams were included in systematic pre-season evaluation at the start of the 2017–2018, 2018–2019 and 2019–2020 sport seasons. Knee isokinetic strength, single leg countermovement jumps, and one leg hop tests were systematically measured. Basketball players who presented previous knee surgery or trauma, patellar cartilage lesions confirmed by imaging or painful sequelae of tibial tuberosity osteochondrosis were not included in the study [22]. Basketball players with current PT were compared to basketball players without knee pain. The evaluation of basketball players consisted of the Victorian Institute of Sport Assessment–Patella (VISA-P) score to measure basketball players’ symptoms and sportive activities [23]. The VISA-P presents moderate-quality evidence in terms of reliability and measurement error but high-quality evidence for construct validity [24]. The PT group was defined by a typical history of pain localized to the lower patellar pole or the tendon, for more than 6 weeks and related to basketball practice, and distinct tenderness on palpation corresponding to the painful area [25,26,27]. The control group was defined by lower limbs free of pain. No ultrasounds or MRIs were carried out because all basketball players were able to train 12 h a week and play in friendly matches at the start of the sport season. Knee isokinetic testing, single leg countermovement jump, and one leg hop test were always performed in the same order. The right side was tested first, arbitrarily, to eliminate results variability due to the variation of fatigue caused by previous tests. All subjects received standardized instructions and the examiner demonstrated the jumps before the tests. All performance data were anonymized before analysis to ensure players’ confidentiality after receiving their oral consent. The study was declared to the Research Department of the University Hospital and approved by the local ethics committee (Comité Nantais d’Ethique en Médecine du Sport) under ethical committee registration CNEMS-2021-003. The study was in compliance with the declaration of Helsinki [28].

### 2.2. Isokinetic Assessments

The tests were performed at the start of the sport seasons with a Humac^®^ isokinetic dynamometer (Medimex, Sainte-Foy-lès-Lyon, France) at the Sports Medicine department of the University Hospital of Nantes. The isokinetic dynamometer responds to movements and variations of torque of the athlete using a closed loop control based on four subsystems: control–command, drive, mechanisms and measurement [29]. The control–command system corresponds to the operator–machine interface to set each mode of exercise performed by the athlete. The drive system consists of an electromechanical motor which provides resistance load, connected to the mechanism system through a reducer. The driver is a power amplifier that manages and provides the voltage and electric current needed to the motor, using the reference in the control-command system. The mechanism system executes the patient–machine interface that allows the athlete to perform the evaluation in a comfortable posture, permitting the isolation of the work of the extensors or the flexors muscle group. The measurement system allows torque measurement from the motor electric current. Speed and position sensors are effective by encoder models.

Isokinetic evaluation consists of performing muscular contraction at a predefined angular speed that remains constant during the range of motion except on initial (acceleration phase) and final stages (deceleration phase). The acceleration and deceleration phases are controlled by the informatics pilot of the angular speed to avoid velocity overshoot [30]. The isokinetic dynamometer responds in form of resistance directly proportional to the force exercised by the athlete during the entire range of motion of the knee joint.

The isokinetic tests were performed as previously described by Dauty et al. [31]: Isokinetic tests were preceded by an ergocycle warm up of 10 min. The sitting position was defined with a hip angle of 85°. The knee lateral condyle and the mechanical axis of the dynamometer were aligned. The players were stabilized with belts. The knee range of motion was defined at 100°. Torque was gravity corrected, and the dynamometer was calibrated monthly. Gravity correction corresponds to a specific model of gravitational moment based on a sine function. The gravitational moment is determined by the weight of the limb and dynamometer attachment. A single passive data point can also determine the specific model established by the constructor to determine the gravity correction [32].

Three submaximal movements followed by two maximal movements were initially performed in order to become familiar with the isokinetic movements. Subsequently, the basketball players were tested over three concentric repetitions at 60°/s of angular speed, followed by five concentric repetitions at 180°/s. Between the two series, players had a 30-s recovery period. During the test, verbal encouragement and visual feedback were provided. The same sports physician conducted all the tests [15,31].

Absolute and relative concentric isokinetic knee torques (in relation to bodyweight) were used. The Limb Symmetry Index (LSI) was calculated for knee extensors and flexors at the two angular speeds (60 and 180°/s). For the PT group, the LSI was calculated by relating the PT knee side to the non-PT knee side. For the control group, the LSI was always calculated to obtain a value ≤ 1, so that leg dominance [25] would not be taken into account [33]. A moderate relative reliability of 0.90 was established for isokinetic quadriceps and hamstring strength by intra-class correlation coefficient [34].

**Figure 1:**
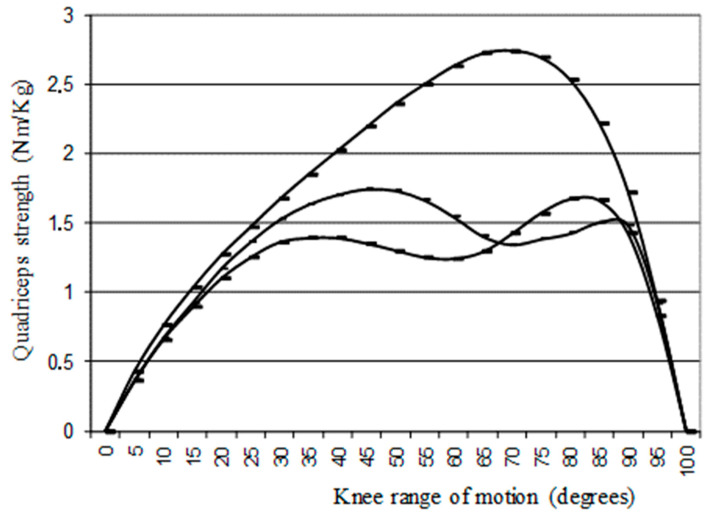
Normal “inverse U curve” and I and II type “camel’s back curve” observed at 60°/s isokinetic angular speed, adapted from Dauty et al. 2019 [31].

A qualitative isokinetic double-humped curve observed during quadriceps strength assessment (only at slow angular speed of 60°/s), referred to as a “camel’s back curve”, was identified (Figure 1) [31].

### 2.3. Jumping Assessments

The single leg countermovement jump (CMJ) was measured using an Abalakov belt (precision of 1 cm). Players had to stand on one leg, descend into a countermovement, and rapidly extend the standing leg to jump vertically, as high as possible [35]. The subjects performed three maximal jumps for each leg with free arms, starting with the right leg and then alternating between the both sides. If subjects’ jump performances kept increasing after three jumps, additional trials were carried out until the height of the jump stopped increasing. For each leg, we used the highest jump for data analysis and we considered it in absolute and relative distance (in relation to bodyweight). The CMJ has been found to be reproducible (ICC of 0.98 [0.96–0.99]) [36]. The unilateral CMJ LSI was calculated with the same method used for the isokinetic LSI.

The one leg hop test was measured with a tape meter (precision of 1 cm). All the basketball players were asked to jump as far as possible with free arms, taking off and landing on the same foot and keeping their balance on this foot for 2 s after landing. The best jump length for each leg was used for data analysis and considered in term of absolute and relative distance (in relation to bodyweight). The one leg hop test had previously been found to be reproducible (ICC of 0.91 [0.83–0.97]) [37]. The hop LSI was calculated with the same method used for the CMJ and the isokinetic LSI.

### 2.4. Statistical Analysis

Statistical analysis was realized with SPSS 23.0^®^ software (Armonk, NY, USA). Quantitative parameters were presented as mean and standard deviation and qualitative parameters as frequency. The Kolmogorov–Smirnov test was used to assess the normality of the tested parameters. A first statistical analysis was performed taking into account the basketball players as unit [38]. Student’s *t*-tests were performed to compare qualitative data of basketball player groups, with and without PT, after verification of variances by the Levene test. χ^2^ tests were used to compare qualitative parameters. Spearman correlations (r) were performed to establish links between knee muscle strength LSI and jump LSI because the variables were not normally distributed. A second statistical analysis was performed to compare leg-to-leg taking the knee as unit [38], i.e., to compare the legs with PT (*n* = 24) to those without PT (*n* = 100). Comparisons were performed using Student’s t-test for relative quantitative parameters. Spearman correlations (r) were performed to establish links between relative knee isokinetic strength and relative jump performances. Statistical significance was established at *p* < 0.05. Two binary stepwise ascendant logistic Wald regressions were performed including predictor parameters with a probability ≤ 0.10 to identify basketball players with PT or knees with PT. Results were expressed as odds ratios (ORs = probability/(1-probability)) [39]. The probability for the first model was the development of PT for a basketball player and for the second model the development of PT for a knee. Because of the inclusion of continuous and categorical variables, the estimation of the ORs was performed as exponential of the coefficient *Ɓ* of the logistical regression [38]. To determine if the data fitted the model well, we used the Hosmer–Lemeshow test. To assess if the model was well adjusted, we used Cox–Snell and Nagelkerke R-squares (% of the variance explained by the predictors). The ROC curve was established to determine sensitivity and specificity of continuous variables included in models. The ROC curve area interpretation was excellent (>0.9), good (0.8–0.9), fair (0.7–0.8), poor (0.6–0.7) or failed (0.5–0.6) [40]. Youden index was used in conjunction with ROC analysis to find the optimum cut-off for numeric predictor parameters [41,42]. The cut-off chosen for the value of test gave equal weight to false positive and false negative values for the groups with and without PT.

## 3. Results

### 3.1. Participants

Eighty-five professional basketball players were evaluated at the start of the season. Eleven were excluded because of a previous knee surgery (six cases) or patellar cartilage damage (five cases). Twelve basketball players were removed from the study because of a bilateral PT, biasing the calculation of isokinetic and jump LSI.

Data of 62 professional basketball players were finally assessed. Players practiced at the Pro A level (22 players), Pro B level (26 players) and National 1 level (14 players). They were 25.0 ± 4.0 years old, had a mean bodyweight of 96.0 ± 11.0 Kg and a mean body-height of 198.0 ± 8.0 cm (Body Mass Index: 25.5 ± 2.2 Kg/m^2^). The mean number of basketball seasons practiced at a professional level was 5.4 ± 4.6 years. Twenty-two players were point or shooting guards (Number 1 or 2), 28 small or power forward (Number 3 or 4) and 12 centres (Number 5).

Twenty-four players out of 62 (38.7%) declared unilateral patellar tendon pain (10 Pro A, 8 Pro B and 6 N1). Nine cases reported knee pain in the right side and 15 in the left side. The pain location was the lower pole of the patella in 16 cases and the tendon in eight cases. The PT history started on average 4.0 ± 3.0 years ago.

### 3.2. Comparison of Basketball Player Groups with and without Patellar Tendinopathy

No significant difference was found between the two groups concerning age, bodyweight, and body-height (Table 1). A trend towards a difference was found for the BMI (*p* = 0.06). The distribution of PT according to position on the field and practice level was not different (Table 1). However, the PT group had been practicing basketball at a professional level for a longer time than the group of players without PT (6.5 ± 4.0 vs. 4.5 ± 3.0 years; *p* = 0.04). The VISA-*p* score was significantly lower in the PT group compared to the non-PT group (81 ± 16 vs. 94 ± 10; *p* = 0.0001).

The PT group presented a quadriceps LSI significantly lower than the non-PT one, while the hamstring LSI was not different (Table 2). The CMJ LSI of the PT group trended lower while the hop LSI was not different (Table 2). No link was found between quadriceps and hamstring LSI at 60 and 180°/s with the CMJ or the hop LSI.

### 3.3. Prediction of Basketball Players with Patellar Tendinopathy

The best model to predict the absence of PT in basketball players included quadriceps LSI at 60°/s (QLSI60) and VISA-P score after exclusion of BMI, quadriceps LSI at 180°/s, CMJ LSI and professional basketball practice duration. The percentage of correct classification by hazard was 61.3%, and the prediction by the model was 72.6% [ORs QLSI60: 0.01 (95%CI: 0.001–0.06); *p* = 0.006 and ORs VISA-P score: 0.94 (95%CI: 0.90–0.99); *p* = 0.03]. The ROC curve area for the QLSI60 was 0.813 [95%CI: 0.694–0.931] and the sensitivity and specificity were 78.9% and 79.2%, respectively, if the cut-off of the quadriceps LSI at 60°/s was fixed at 89% according to the Youden index. The ROC curve area for the VISA-P score was 0.785 [95%CI: 0.667–0.903] and the sensitivity and specificity were 73.7% and 75%, respectively, if the cut-off of the VISA-P score was fixed at 94% according to the Youden index.

### 3.4. Comparison between Legs with Patellar Tendinopathy and those without

Relative quadriceps strength was significantly lower on the leg side with PT than on the one without, explaining a strength ratio (H/Q) significantly higher in the legs with PT. Jump performances were not different (Table 3).

A moderate link was established between relative jump performance and relative isokinetic strength of the quadriceps and the hamstring. No link was found between jump performances and hamstring-to-quadriceps ratios (Table 4).

A camel’s back curve was identified during quadriceps isokinetic assessment at 60°/s in 58% of the legs with PT vs. 7% of the +healthy legs (F = 36; *p* = 0.0001; sensitivity of 58.3% and specificity of 93%). The proportion of type I and II camel’s back curves is described in Table 5.

### 3.5. Prediction of Patellar Tendinopathy

The best model to predict a knee without PT included relative quadriceps strength at 60°/s and the camel’s back curve, after exclusion of relative quadriceps strength at 180°/s. The percentage of correct classification by hazard was 67.5%, and the prediction by the model was 80.5% [ORs relative Q60: 0.25 (95%IC: 0.09–0.68); *p* = 0.007 and OR camel’s back curve: 0.08 (95%IC: 0.03–0.19); *p* < 0.001]. The ROC curve area for the relative strength of quadriceps at 60°/s was 0.748 [95%CI: 0.666–0.830] and the sensitivity and specificity were respectively 67.3% and 68%, if the cut-off of the relative quadriceps strength at 60°/s was fixed at 2.35 Nm/Kg according to the Youden index.

## 4. Discussion

When PT was clinically diagnosed in professional basketball players at the start of the sport season, the VISA-P score and isokinetic quadriceps strength were low and often associated with a camel’s back curve. However, single leg countermovement jump and one leg hop test performances were preserved. Professional basketball level and field position had no influence. Professional basketball practice duration was higher compared to basketball players without PT, and there was a trend toward a difference for the BMI. When these parameters were analyzed alone, high BMI seemed to be associated with PT but was not a risk factor of PT [2,10,11,14,43], whereas intensity of basketball practice was considered an independent risk factor [14].

Concerning knee strength, no association was found between PT and hamstring isokinetic strength or hamstring-to-quadriceps strength ratios. This finding had already been reported, although no inhibition of hamstring strength was associated with PT [13,44]. These parameters were not favorable to detect PT at the start of the sport season. Concerning quadriceps strength, the relationship with PT remains unclear because of studies using different methods with insufficient volume [22,44,45]. Krauss and al. found no difference in quadriceps strength in a group of female runners, which was significantly different from a male professional basketball player population [45]. Kujala and al. showed a significant lower relative quadriceps strength in a group of high-level athletes with PT compared to a control group (4.06 vs. 4.48 Nm/Kg) [44]. Scattone et al. also reported a lower normalized quadriceps strength (but not significantly lower) in a PT group compared to a control group (0.98 vs. 1.16 Nm/Kg/m) [22]. Kabacinski et al. also reported in female volleyball players lower isokinetic quadriceps strength at 60°/s in case of stage-1 PT [46].

It has been previously shown that the highest jump reached during several jump procedures, such as the countermovement jump, is preserved in cases of PT in volleyball players [25,47,48]. Vertical jump distance is also preserved in elite male junior basketball players with PT [49]. The probable explanation was that the players were all able to practice competitively at the time of the jump assessment despite a VISA-P score less than 80 points [49]. However, from a kinematic point of view, Pietrosimone et al. showed that athletes with symptomatic PT had a reduced knee flexion angle throughout the stance phase, reduced internal knee extension momentum and reduced patellar tendon force in early stance during double limb jump-landing compared to healthy controls [50]. In fact, jump performance requires a more complex set of muscles than quadriceps muscle strength alone, despite a relationship between jumps and quadriceps strength. Indeed, we have found a moderate relationship (r > 0.300) when the knee is considered for analysis. No study has reported these correlations in a group of athletes with PT. In healthy volleyball players and elite female basketball players, significant correlations were reported (r > 0.700) [51,52]. Because correlations were higher with isokinetic quadriceps strength than with hamstring strength, knee extensors should be considered of greater importance to jumps than knee flexors. Indeed, the quadriceps muscles are working agonistically at the knee joint level during the impulsion phase, exerting much of the strength required for jumps [51].

To our knowledge, only two studies have compared muscular knee strength and jump performances in a PT group vs a control group, neither one among professional basketball players [43,53]. Comparison between these two studies may be difficult because they refer to different sports populations. Gaida et al. [53] studied a national female basketball population aged 20, and Crossley et al. [43] a female and male athletes’ population playing 5 different sports in competition (basketball, netball, volleyball, soccer and tennis). Moreover, these two studies are limited because they are representative of very small groups (*n* < 20), including a group with bilateral PT. However, Crossley et al. found a lower normalized isometric quadriceps strength and hop test (137 cm vs. 160 cm) in case of unilateral PT [43]. In these studies, the participants’ long period of pain contributed to muscle inhibition, inducing muscle atrophy. Reduced strength was reported to explain reduced hop performance, or vice versa. Gaida et al. found results similar to ours [53]: a trend toward difference in isokinetic quadriceps LSI at 180°/s between the unilateral PT group and the control group was reported in an eccentric mode, while vertical jump performances were not different. For these authors, a decrease in eccentric strength may initially protect the tendon from excessive stress, but a decrease in controlled muscle strength may ultimately be detrimental for the tendons. As such, the interaction between isokinetic quadriceps strength and jump performance is difficult to understand in cases of PT.

However, lower quadriceps strength is a consequence of PT. Indeed, Witvrouw et al. showed no difference in absolute isokinetic strength before the onset of PT in 19 subjects in a prospective predictive study during a 2-year follow-up of a 19 year-old athletic population (*n* = 138) [13]. In cases of reported PT, there was already a quadriceps isokinetic strength difference of 25% at 60°/s [15]. In 2019, a study showed an association with a camel’s back curve observed at 60°/s of angular speed [31], which was an additional argument for the importance of PT. In fact, this curve was described for the first time by Ayalon et al. in 2002 during painless active isokinetic open chain extension of the knee [54] and Dauty et al. have reported this anomaly in 81% of professional basketball players with a PT history [31]. Two types were distinguished depending on whether the first peak was greater than the second (type I) or the reverse (type II). The “camel’s back curve” could be explained by a protective inhibition to limit knee stress due to patellar tendon tension as a circuit breaker of the quadriceps contraction to protect the muscular–tendon unit [55]. In the present study, this curve was significantly observed in 58% of legs with PT and 7% of legs without PT. The specificity was also different from the 100% specificity described in the original study by Dauty et al. [31]. A possible explanation is that some basketball players may have signaled only unilateral PT while they were in reality suffering from controlled knee pain on both sides.

In cases of PT, compensatory strategies have been observed, particularly during the horizontal landing phase after forward acceleration in a hop test [56]. An increase in hip flexion, a higher hip extension velocity, an increase in knee flexion and a higher knee extension velocity were described. A decreased ankle dorsiflexion coupled with an eccentric contraction of the calf muscle was also reported to absorb lower limb force when landing from a jump [48]. In basketball players with PT, Siegmund et al. have confirmed compensatory strategies with a kinematic analysis [57]. The countermovement jump was preserved compared to a control group (64 cm vs. 63 cm), because of compensation with an increase in the hip flexion and a decrease in the hip acceleration. During landing in a countermovement jump, a significant reduction in knee acceleration was also observed.

Consequently, in basketball players able to play, jump performances are preserved despite low relative quadriceps isokinetic strength due to PT. This is due to compensatory strategies. However, jump performances may be altered if the basketball players are not able to practice because of unbearable patellar tendon pain. In fact, playing basketball with tendon pain is common due to a “positive culture” of PT in jumping sports [17]. Athletes with PT typically continue their practice, despite the presence of pain [22].

## 5. Limitations

PT was clinically diagnosed without exploration of the tendon structure by ultrasound [58]. However, the possibility of false positive PT was limited because of the inclusion and exclusion criteria and the fact that the PT players were only male, aged 26 and practicing for more than 6 years at a professional level. Recently, it has been demonstrated that pathological patellar tendons detected via imaging were not significantly associated with the development of symptoms (RR = 1.8, 95%CI: 0.9–3.7), and a previous history of PT was the strongest predictor for the development of symptoms (patellar RR = 3.7 95%CI: 2.2–6.1) [1].

The VISA-P score of 81 points could appear high because it was not less than 60 points [43], which is explained by the ability of all our basketball players to play at the start of the season, similar to the PT population examined by Gaida et al. (VISA-P score: 83 points) [53].

Only male professional basketball players with or without unilateral PT were included in this study. The extrapolation of these findings to female athletes, to other sports, or to younger basketball players, and to basketball players with bilateral PT should be done with caution. It seems that athletes with bilateral patellar tendinopathy are a particular population with more morphological anomalies and weaker VISA-P scores [43,53].

The strength assessment performed in this study was concentric and might not closely reflect the torque generation of the knee muscles during jump-landing sports. The deceleration phase of a jump corresponds to an eccentric muscle mode of contraction [53].

Finally, due to the cross-sectional design, the link between knee strength, functional jumps and PT may be clearer. Low quadriceps strength associated with a camel’s back curve is a consequence of PT [31].

## 6. Conclusions

VISA-P score and isokinetic knee assessment were very instructive to identify professional basketball players suffering from patellar tendinopathy at the start of a sport season. The predictive model included low quadriceps LSI at 60°/s and low VISA-P score or low relative quadriceps isokinetic strength at 60°/s associated with a camel’s back curve. Cut-offs to identify professional basketball players with patellar tendinopathies were 89% for the quadriceps LSI at 60°/s, 94 points for the VISA-P score and 2.35 Nm/Kg for the relative quadriceps strength at 60°/s, associated in 58% of cases with a camel’s back curve. Moreover, single-leg countermovement jumps and one leg hop tests were not favorable for this objective when basketball players were able to practice competitively. This point highlights that field and functional tests are insufficient to detect patellar tendinopathies and therefore laboratory evaluations in professional basketball players are more relevant at the start of the sport season. Athletic trainers and sport medicine professionals who provide medical care to male professional basketball players should consider assessing the patellar tendon during preseason with a protocol including VISA-P score and isokinetic quadriceps assessment at 60°/s, to identify a camel’s back curve as a consequence of patellar tendinopathy. Therapeutic programs to improve modifiable parameters such as quadriceps strength could be proposed, with isokinetic follow-ups, to allow players to continue to practice with tolerable tendon pain, without interfering with their function in training or competition later during the sport season.

## Figures and Tables

**Table 1:** Comparison of professional basketball players according to the presence or absence of patellar tendinopathy.

	Players with Patellar Tendinopathy (*n* = 24)	Players without Patellar Tendinopathy (*n* = 38)	*p*
Age (Years)	26 ± 5	24 ± 4	0.13
Weight (Kg)	97 ± 12	95 ± 12	0.57
Height (cm)	196 ± 7	199 ± 9	0.36
BMI (Kg/m^2^)	25.1 ± 0.2	24.1 ± 0.2	0.06
Professional practice duration (year)	6.7 ± 4.1	4.6 ± 3	0.03
Position:			0.90 *
Point and Shooting guard	9 (37.5%)	13 (34.2%)	
Small and Power forward	10 (41.7%)	18 (47.4%)	
Center	5 (20.8%)	7 (18.4%)	
Sport Level:			0.54 *
Pro A	10 (41.7%)	12 (31.6%)	
Pro B	8 (33.3%)	18 (47.2%)	
National 1	6 (25%)	8 (21.1%)	
VISA-P score	81 ±16	94 ± 10	0.002

Abbreviations: BMI: Body Mass Index; VISA-P score: Victorian Institute of Sport Assessment- Patella score. * χ^2^ test.

**Table 2:** LSI Comparison between basketball players with and without patellar tendinopathy.

	Basketball Players with Patellar Tendinopathy (*n* = 24)	Basketball Players without Patellar Tendinopathy (*n* = 38)	*p*
Q60 LSI (%)	81 ± 11	91 ± 8	0.001
Q180 LSI (%)	87 ± 10	92 ± 6	0.01
H60 LSI (%)	91 ± 6	89 ± 7	0.31
H180 LSI (%)	92 ± 4	91 ± 8	0.59
CMJ LSI (%)	86 ± 10	90 ± 6	0.08
Hop LSI (%)	96 ± 3	94 ± 5	0.13

Abbreviations: Q60 LSI: Quadriceps Limb Symmetric Index at 60° of isokinetic angular speed; H: Hamstring. CMJ: Countermovement Vertical Jump.

**Table 3:** Absolute and relative knee strength and jump performances of legs with patellar tendinopathy and those without.

	Patellar Tendinopathy Legs (*n* = 24)	Legs without Patellar Tendinopathy (*n* = 100)	*p*
Q60 (Nm)	212 ± 38	243 ± 49	0.0001
Q180 (Nm)	169 ± 32	177 ± 32	0.17
H60 (Nm)	156 ± 28	153 ± 28	0.66
H180 (Nm)	125 ± 34	120 ± 21	0.27
Q60 (Nm/Kg)	2.14 ± 0.42	2.54 ± 0.44	0.0001
Q180 (Nm/Kg)	1.70 ± 0.31	1.84 ± 0.26	0.003
H60 (Nm/Kg)	1.56 ± 0.25	1.61 ± 0.26	0.28
H180 (Nm/Kg)	1.25 ± 0.31	1.26 ± 0.20	0.90
H/Q60	74 ± 1 4	64 ± 12	0.0001
H/Q180	74 ± 16	69 ± 12	0.03
Absolute CMJ test (cm)	42.7 ± 6.3	41.2 ± 7	0.19
Relative CMJ test (cm/Kg)	0.43 ± 0.08	0.43 ± 0.09	0.89
Absolute Hop test (cm)	228 ± 20	226 ± 23	0.69
Relative Hop test (cm/Kg)	2.32 ± 0.39	2.40 ± 0.42	0.24

Abbreviations: Q60: Quadriceps strength at 60° of isokinetic angular speed; H: Hamstring; H/Q: Conventional Hamstring-to-Quadriceps ratio; CMJ: Countermovement Vertical Jump.

**Table 4:** Spearman’s correlation between relative isokinetic strength and jump performances in legs with patellar tendinopathy and those without.

		Q60/Kg	Q180/Kg	H60/Kg	H180/Kg	H/Q60	H/Q180	CMJ/Kg
PT Legs (*n* = 24)	CMJ/Kg	0.375 **	0.372 **	0.304 *	0.154	−0.288	−0.280	1
	Hop/Kg	0.313 *	0.361 **	0.324 *	0.209	−0.108	−0.145	0.818 ***
Legs without PT (*n* = 100)	CMJ/Kg	0.203 *	0.260 **	0.343 ***	0.339 ***	0.151	0.157	1
	Hop/Kg	0.217 *	0.159	0.309 **	0.205 *	0.048	−0.092	0.686 ***

Abbreviations: Q60/Kg: Relative Quadriceps strength at 60 degrees per second per Kilogramme; H: Hamstring; CMJ/Kg: Relative Countermovement Jump per kilogramme; Hop/Kg: Relative Hop per kilogramme; PT: Patellar Tendinopathy. * *p* < 0.05; ** *p* < 0.01; *** *p* < 0.001.

**Table 5:** Proportion of isokinetic camel’s back curve as a function of leg with or without patellar tendinopathy.

Camel’ Back Curve	PT Legs (*n* = 24)	Legs without PT (*n* = 100)	χ^2^ test
No	10 (41.7%)	93 (93%)	F = 9.6; *p* = 0.008
Type I	7 (29.2%)	4 (4%)	
Type II	7 (29.2%)	3 (3%)	

## Data Availability

The data presented in this study are available on request from the corresponding author. The data are not publicly available due to ethical reasons.
